# Mending the Valve, Burdening the Kidney: A Case of Renal Hemosiderosis With Mitral Valve Repair

**DOI:** 10.1155/crin/2777348

**Published:** 2025-03-19

**Authors:** Sadikshya Bhandari, Tenzin Tamdin, Raymond Raut

**Affiliations:** ^1^Department of Internal Medicine, Danbury Hospital/Nuvance Health, Danbury, Connecticut, USA; ^2^Department of Nephrology, Danbury Hospital/Nuvance Health, Danbury, Connecticut, USA

## Abstract

Renal hemosiderosis, marked by the buildup of hemosiderin in the renal cortex due to chronic intravascular hemolysis, can lead to acute kidney injury. Iron deposited may exacerbate renal damage through processes like lipid peroxidation and free radical formation, impacting cellular function and precipitating renal disease. Although seen commonly with chronic intravascular hemolysis in the setting of sickle cell anemia, thalassemia, and in the setting of prosthetic cardiac valves. While acute renal failure following prosthetic cardiac valve placement is relatively uncommon, some cases of renal injury in the setting of mechanical hemolysis have been reported, in the postsurgical period, typically within 2 weeks. In this case report, we present a 67-year-old male patient, following mitral valve repair, who developed anemia within 2 weeks of the repair. Evaluation with echocardiography did not initially show findings of worsening mitral regurgitation, however, his anemia and kidney function worsened over the next 6 months. Further evaluation, during that time showed worsening mitral valve regurgitation in repeated echocardiography, with renal hemosiderosis confirmed via kidney biopsy, revealing glomerulosclerosis with hemosiderin deposits. Due to persisting anemia and worsening kidney function, the patient is being planned for a mitral valve replacement. The potential recovery of renal function remains uncertain. Despite the common occurrence of intravascular hemolysis following prosthetic valve placement or valve repair, only a few cases of acute renal failure have been reported. In these cases, severe hemolytic anemia developed shortly after surgery, within 2 weeks, with increased levels of creatinine, even reported as high as 8.2 mg/dL and renal biopsy revealed acute tubular injury and widespread tubular hemosiderosis, resembling the findings in the index case. Diagnosis depends on the evaluation of anemia, showing signs of hemolysis, with echocardiology findings of worsening valvular abnormalities, and kidney biopsy findings showing hemosiderin deposits. Treatment strategies encompass the management of anemia alongside considerations for either mitral valve replacement or repair. This case underscores the importance of considering the possibility of renal hemosiderosis, associated with mitral valve repair. The delay in diagnosis observed in this case is not uncommon, and timely recognition becomes pertinent to prevent irreversible renal injury and improve long-term outcomes.

## 1. Introduction

Hemosiderosis is the excessive accumulation of hemosiderin, a form of iron storage complex composed of partially digested ferritin and lysosome derived primarily from the breakdown of erythrocytes. Renal hemosiderosis is characterized by the deposition of hemosiderin in the renal cortex secondary to chronic mechanical intravascular hemolysis from valvular disease, paroxysmal nocturnal hemoglobinuria, or refractory anemia requiring repeated blood transfusion [1]. Although traumatic intravascular hemolysis is considered a complication of valvular heart disease or valve replacement surgery, renal hemosiderosis secondary to intravascular hemolysis in the setting of mitral valve repair has rarely been reported [1, 2].

## 2. Case

The index patient, a 66 year old male, with extensive medical history, including mitral insufficiency in the setting of mitral valve prolapse, hypertension, hyperlipidemia, peripheral arterial disease, chronic kidney disease (CKD) stage III, chronic anemia, former smoker and history of adenomatous polyps in the colon. In the latter half of 2022, the patient began experiencing fatigue and dyspnea on exertion. Further investigation, including echocardiography (11/8/2022), revealed moderate prolapse of the anterior leaflet with a flail segment of the mitral valve, accompanied by severe regurgitation, despite which he had an ejection fraction of 60%–65% and no signs of pulmonary hypertension. The patient underwent a successful surgical mitral valve repair in January of 2023. However, the postoperative period was marked by complications, including respiratory failure, atrial fibrillation, hyponatremia, and hyperkalemia.

Ten days postsurgery, he presented with lower extremity edema and later complained of left-sided flank pain. Subsequent evaluation identified a ureteropelvic junction stone, leading to acute kidney injury. Urological interventions were performed, including a left double-J ureteral stent placement and renal extracorporeal shockwave lithotripsy. Despite these interventions, he developed dysuria, leading to a course of ciprofloxacin for urinary tract infection, and later Bactrim for recurrent UTI.

**Figure 1:**
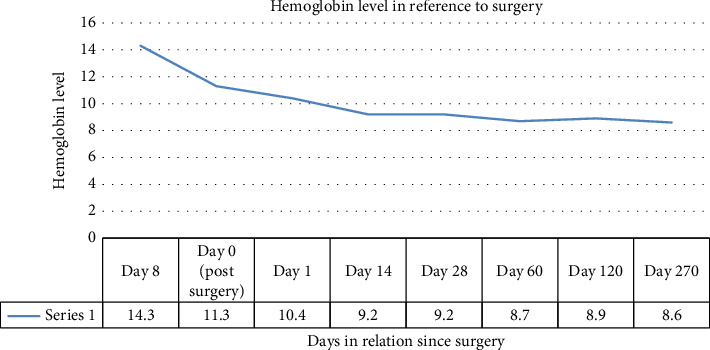
Hemoglobin level in relation to surgery.

As seen in Figure 1, his baseline hemoglobin was at 14.3, with hemoglobin following surgery at 11.3, there was further consistent drop in hemoglobin in the following weeks, with another one point drop seen in 2 weeks. Up to this point his anemia was considered in the setting of blood loss during surgery. He had required no transfusions. He received no iron or folic acid supplements.

During this period, he did have a routine colonoscopy done, revealing the presence of adenomatous polyps and internal hemorrhoids in the colon. Despite an improvement in shortness of breath over the months, the patient continued to experience severe fatigue, poor appetite, and a weight loss of 20 pounds.

Repeat echocardiography, 2/8/2023, showed an annuloplasty ring in the mitral valve, with moderately thickened anterior leaflet, and trace regurgitation. Mildly dilated left atrium. Despite this anemia persisted, with hemoglobin levels ranging from 8–9, below his baseline of 10–11, even three months postsurgery. Platelet levels were also low, as seen in Table 1.

The patient was started on ferrous sulfate for suspected iron deficiency anemia, and a further evaluation, including a peripheral smear and iron panel, showed ferritin of 58. He had normal folic acid and vitamin B12 levels. Although tick panel was negative, he was treated with doxycycline for sinusitis for 2 weeks by his ENT physician. He was also started on folic acid by hematology. Iron tablets continued with no improvement in anemia.

With persistent anemia as well as continued dyspnea, hematology referral followed, with tests such as LDH, haptoglobin, Coombs test, PT, PTT, fibrinogen, SPEP, IFE, FLC, and quantitative Ig, EPO. The patient was found to have high LDH, and low haptoglobin, with concern for hemolytic anemia. The peripheral blood smear examination revealed macrocytic normochromic red blood cells with mild anisopoikilocytosis, including occasional schistocytes (one to three per high-power field), rare target cells, and rare elliptocytes. Platelets showed no significant variation in size or clumping. Lymphocytes consisted of a heterogeneous population of lymphoid cells, while neutrophils exhibited no significant morphological abnormalities. No blasts or parasites were observed in the examination. Other results indicated no monoclonal gammopathy, and normal immunoglobulins, as seen in Table 2.

The patient was further referred to nephrology due to worsening kidney function and increasing creatinine levels. The patient's peripheral blood smear also showed schistocytes, although minimal with concern for microangipathic hemolytic anemia. Following which, additional investigations included repeat echocardiography, immunology workup (including ADAMTS13), and plans for a renal biopsy.

Repeat echocardiography 11/03/2023, showed thickened mitral valve leaflets, with an annuloplasty ring present, two jets of mitral regurgitation, moderate, with anterior leaflet prolapse was noted.

**Figure 2:**
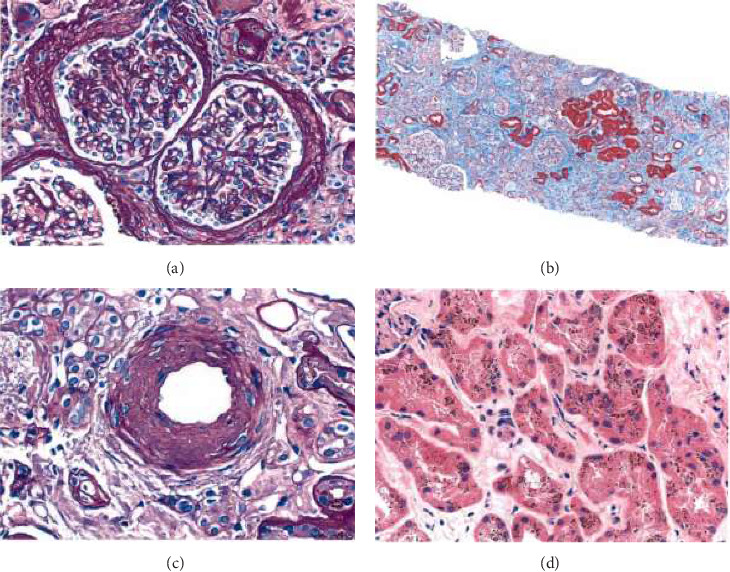
Renal biopsy, (a) focal global glomerulosclerosis, moderate diffuse ischemic glomerular changes, tubular atrophy, interstitial fibrosis, (b) scarred cortex, (c) arteriolar sclerosis, (d) hemosiderin deposits.

The biopsy revealed focal global glomerulosclerosis, moderate diffuse ischemic glomerular changes, tubular atrophy, interstitial fibrosis, arterial and arteriolar sclerosis, and hemosiderin deposits in proximal tubular cells consistent with renal hemosiderosis, likely related to macroangiopathic hemolytic anemia postmitral valve repair, as seen in Figure 2.

Following the diagnosis, the patient was referred back to cardiology and the patient is currently being planned for a complete mitral valve replacement to ensure the best possible outcome for the patient.

## 3. Discussion

Mitral valve repair, in primary mitral regurgitation, although challenging, has around an 80% success rate for avoiding reoperation and a 60% success rate for preventing recurrent significant mitral regurgitation over 15–20 years [3–5]. Older patients are seen to have better results [6]. In our case report, the patient underwent mitral valve repair for mitral valve prolapse complicated by severe mitral regurgitation. Recurrent mitral regurgitation is a common complication after primary mitral valve repair, which can lead to mechanical hemolysis, however, in the afore-mentioned case was further complicated by hemosiderin deposits in the kidneys leading to kidney injury [1, 7]. Acute renal failure due to intravascular hemolysis following prosthetic cardiac valve placement has been reported infrequently [8].

Hemosiderin is identified as a granular yellow-brown degradation product representing an iron-storage complex. Hemosiderin is produced through the breakdown of hemoglobin or an abnormal metabolic pathway involving ferritin [9, 10]. Iron deposition occurs when red blood cells are destroyed, releasing hemoglobin into the bloodstream. The hemoglobin forms a complex with haptoglobin and hemopexin, preventing it from passing through the glomerulus. With the saturation of these systems, free hemoglobin circulates briefly, dissociating into alpha beta dimers that can pass through the glomerulus. In the proximal tubule, reabsorbed hemoglobin releases iron, incorporating it into ferritin and hemosiderin. Prolonged hemolysis may lead to renal siderosis. Iron-laden tubular cells shed into the urine increase urine iron concentration, with ferritin and hemosiderin detectable. 1ron's potential stimulation of lipid peroxidation and free radical formation may impact cellular function [9, 11].

Despite the common occurrence of intravascular hemolysis following prosthetic valve placement or valve repair, only a few cases of acute renal failure have been reported. In these cases, severe hemolytic anemia developed shortly after surgery, within 2 weeks, with increased levels of creatinine, even reported as high as 8.2 mg/dL and renal biopsy revealed acute tubular injury and widespread tubular hemosiderosis, resembling the findings in the index case [1, 8, 12, 13].

Until now, most cases of hemolytic anemia with renal hemosiderosis are not due to the mechanical shear effect from the valve. The classic one is PNH, but it was also seen in SCD, HS thalassemia, transfusion reaction, and auto-immune hemolytic anemia [14]. Our patient's percutaneous renal biopsy showed focal global glomerulosclerosis, moderate diffuse ischemic glomerular changes, tubular atrophy, interstitial fibrosis, arterial and arteriolar sclerosis, and hemosiderin deposits in proximal tubular cells consistent with renal hemosiderosis. Based on MVR with lab findings suggestive of hemolytic anemia, AKI, and renal biopsy findings, the patient was diagnosed with macroangiopathic hemolytic anemia and renal hemosiderosis following mitral valve repair. Though renal hemosiderosis may or may not have been an incidental finding. Renal hemosiderosis can be a diagnostic consideration when MRI cindery reveals abnormally low renal cortical signal in T1W and T2W sequences [15].

Very few studies have addressed the prognosis and treatment of hemolytic anemia secondary to valvular dysfunction and subsequent renal hemosiderosis. As per the European Society of Cardiology Guidelines, valve replacement or re-operation was recommended in patients with clinically significant hemolytic anemia [16]. Our patient also has significant hemolytic anemia secondary to mechanical shear and subsequent renal hemosiderosis. Our patient is currently undergoing evaluation for mitral valve replacement. The treatment in terms of non-severe hemolytic anemia secondary to valve repair is pharmacologic approaches with iron, folic acid, and vitamin B12 for symptomatic relief [12]. In a previous case report, the use of antioxidant N-acetylcysteine has been tried and led to the resolution of hemolysis by improving RBC elasticity [12]. In one of the RCTs, Pentoxifylline 400 mg TID was found to be associated with improvement in mechanical intravascular hemolysis (MIH) in 60% of patients with mitral and aortic valve prostheses [17]. Propranolol has also reduced the severity of hemolysis in 5 patients with severe anemia from prosthetic valves [18]. However, in our patient, mainly the use of ferrous sulfate and folic acid was used with stabilization of the hemoglobin level, but without any improvement. Failure of creatinine level to improve can be best explained by changes noted on the renal biopsy specimen that is, hemosiderin deposit in the proximal tubular cell, and focal global glomerulosclerosis.

This study underscores the importance of diagnostic workup for persistent anemia, despite recent surgery. In this case although worsening hemoglobin levels were considered in the setting of intraoperative blood loss, no improvement in anemia despite iron supplementation, in over 6 months warranted workup. Signs of hemolysis were seen which warranted further workup and definitive treatment of the primary cause of hemolysis. Further, this case report also underscores an unusual presentation of renal hemosiderosis in the setting of macroangiopathic hemolytic anemia, demonstrating the importance of renal workup as well as kidney biopsy which helped us reach a diagnosis. Although routine markers of hemolysis wouldn't be warranted routinely post valvular surgery in but we strongly advocate for further workup for persistent anemia and worsening AKI, while considering the possibility of macroangiopathic hemolytic anemia in a patient with valve repair. There are no other definitive recommendations for medications that would decrease the risk of hemolysis as per the studies mentioned above. Iron, folic acid and vitamin b12 supplementation, in the setting of deficiency would be beneficial. There have been no recommendations for erythropoietin either unless in the setting of deficiency, in CKD. But treatment of the primary cause of the hemolysis would be the principal management strategy in patients. The delay in diagnosis in our case occurred due to the rarity of the presentation as well as alternative explanations for the anemia itself, including perioperative blood loss and iron deficiency.

## Figures and Tables

**Table 1:** Bloodwork done postsurgery in 2023.

Labs	Normal values	Postsurgery	5 months postsurgery	9 months postsurgery
Complete blood counts				
WBC	3.5–10 × 10 (9)	10.9	8.6	4.1
RBC	4.5–5.7 × 10 (12)	3.3	2.8	2.5
HB	13.5–17	10.4	8.9	9
HCT	38–50	29.5	28.7	25
MCV	80–99 FL	88.6	102.5	102.4
MCH	25–34 PG	31.2	31.8	36
MHCH	31–36 g/dL	35.3	31	35.2
RDW	11.5%–15%	13.3	14.5	12.9
Platelet	150–400 × 10 (9) L	88	188	138
LDH	122–222 u/L			1755
Iron panel				
Iron	50–158 μg/dL			110
TIBC	240–450 μg/dL			338
Transferrin saturation	14%–50%			33%
Ferritin	24–370 ng/mL			58
Vitamin b12	230–1245			1154
Folate	> 4 ng/mL			> 20
Coagulation studies				
APTT	25–35.8 s	34.1		30.9
PT	12.2–14.5 s	15.6		13.8
INR	0.91–1.40	1.23		1.06
D dimer	< 500 ng/mL			1890
Fibrinogen	210–480 mg/dL			334
Thrombin time	14–21 s			15.5
ESR				2
Chemistry				
Sodium	135–145 mmol/L	134	136	
Potassium	3.5–5.5	3.7	5.1	
Magnesium	1.6–2.6	2.3		
Phosphorus	2.6–4.7	3.4		
Chloride	97–107 mmol/L	104	106	
Bicarb	22–29 mmol/L	24	22	
Anion gap	10–19 mmol/L	11	8	
BUN	6–23 ng/dL	21	30	
Creatinine	0.67–1.23 mg/dL	1.33	1.63	
eGFR	> 60 mL/min/1.73 m^2^	59	46	
Creatinine clearance		60		
Glucose	70–99 mg/dL	101	94	
Calcium	8.6–10.4 mg/dL		9.3	
LFT				
Total bilirubin	0.02–1.2	1.1	0.9	
Alkaline phosphatase	40–130 IU/L	41	82	
ALT	10–62 IU/L	23	37	
AST	10–50 IU/L	77	61	
Albumin	3.7–5.1		4.1	
Globulin	1.8–3.4		2.1	
AG ration	1.1–2.5		2	
Total protein	6.3–7.9		6.2	
Anti pro bnp	< 299	2418		
Urinalysis				
Urine PH	4.8–8	6		
Specific gravity	1.001–1.035	1.017		
Glucose	Negative	Negative		
Ketones	Negative	Negative		
Blood	Negative	Negative		
Protein	Negative	1+		
Urobilinogen	Negative	Negative		
Nitrite	Negative	Negative		
Leukocyte esterase	Negative	Negative		
WBC	0–2	0–2		
RBC	0–2	3–9		
Cast	0–8 lpf	0–8		
Tumor marker				
PSA	< 4		1.2	
Free PSA			0.3	

**Table 2:** Hematology workup.

Labs	Normal values	Patient's values
Hepatitis viral panel		
Hepatis C		Nonreactive
Hepatitis B (HBs antibody)		< 3.5
Serology		
ANA	1:40	1:160
ANA pattern		Speckled
Centromere antibody		Negative
Chromatin antibody		Negative
dsDNA		Negative
Anti JO1 ab		Negative
Ribosomal ab		Negative
RNP ab		Negative
Smith ab		Negative
Scl 17 ab		Negative
Ssl ab		Negative
SSA		Negative
SSB		Negative
C3 complement	75–180 mg/dL	86
C4 complement	10–40 mg/dL	15
GBM ab		Negative
MPO ab		Negative
Proteinase 3 ab		Negative
Lyme screen		Negative
Ig a	70–400 mg/dL	118
Ig G	700–1600 mg/dL	871
Ig M	40–230	176
Albumin fraction	3.7–5.5 g/dL	4.6
Alpha 1 globulin	0.05–0.22 g/dL	0.18
Alpha 2 globulin	0.55–0.87	0.48
Beta globulin	0.53–1.11 g/dL	0.65
Gamma globulin	0.41–1.18 g/dL	0.69
Protein electrophoresis	No abnormal discrete bands suggestive of monoclonal gammopathy.	
Cardiolipin ab IgM	< 20 u/mL	< 1.5
Cardiolipin ab IgG	< 20 u/mL	< 1.6
Kappa free light chain	0.33–1.94 mg/dL	3.74
Lambda free light chain	0.57–2.63 mg/dL	0.57
Kappa/lambda ratio	0.26–1.65	1.36
Direct anti globulin test		Negative
AHG ig G		Negative
Anaplasma PCR		Negative
Babesia PCR		Negative
LDH		
LDH		1103
Fraction I (heart)	14%–27%	42.5%
Fraction II	29%–42%	39.8%
Fraction III	18%–30%	12.8%
Fraction IV	8%–15%	2.8%
Fraction V	6%–23%	2.1%
Erythropoietin	2.6–18.5 min/mL	20.2
Haptoglobin	30–200	< 10

## Data Availability

Data sharing is not applicable to this article as no new data were created or analyzed in this study.
